# Supplementary material for: Too fast to stay on track? Shorter time to first anti-retroviral regimen is not associated with better retention in care in the French Dat’AIDS cohort
Source: PLoS One. 2019 Sep 6;14(9):e0222067. doi: 10.1371/journal.pone.0222067 (PMC6730866; doi:10.1371/journal.pone.0222067)
Supplement: S1 Table — (DOCX) [file pone.0222067.s002.docx]

S1 Table: Retention rate following geographical location of the care center

| Region | In care at month 12 |
| --- | --- |
| Paris Area | 82.5% |
| North East | 85.8% |
| North West | 83.8% |
| South East | 86.3% |
| South West | 82.9% |
| Overseas | 78.2% |
