# Supplementary material for: Too fast to stay on track? Shorter time to first anti-retroviral regimen is not associated with better retention in care in the French Dat’AIDS cohort
Source: PLoS One. 2019 Sep 6;14(9):e0222067. doi: 10.1371/journal.pone.0222067 (PMC6730866; doi:10.1371/journal.pone.0222067)
Supplement: Attachment — Submitted filename: Response to Reviewers.docx [file pone.0222067.s003.docx]

Dear Editor

Thank you for the interest you showed in our work.

We did our best to answer yours and the reviewer’s queries, and in details:

1. The text was edited to meet PLOS ONE's style requirements
2. A lead author for the “Dat’AIDS Study Group” along with a contact email address has been added and the affiliation of the authors added
3. “Data not shown” has been removed from the text and a supplementary table has been added, along with a brief explicative sentence (line 245)
4. We tried to speculate more on the reasons why very fast ART initiation could be related with poor retention, see additional sentences lines 220-225. As we were truly surprised by this result, we do come short to find an explanation, and are planning to investigate this in depth.

We are willing to make more modifications if necessary.

Sincerely

Lise Cuzin

Martinique University Hospital
